# Protective Effects of Methanolic Extract of *Micromeria frivaldszkyana* (Degen) Velen Against Acetaminophen-Induced Liver Toxicity in Male Wistar Rats

**DOI:** 10.3390/ijms26189112

**Published:** 2025-09-18

**Authors:** Elisaveta Apostolova, Kristina Stavrakeva, Vesela Kokova, Ivica Dimov, Mariya Choneva, Delyan Delev, Ilia Kostadinov, Ilia Bivolarski, Maria Koleva, Rumen Mladenov, Plamen Stoyanov, Anelia Bivolarska

**Affiliations:** 1 Department of Pharmacology, Toxicology, and Pharmacotherapy, Faculty of Pharmacy, Medical University of Plovdiv, Vasil Aprilov Str. 15A, 4002 Plovdiv, Bulgaria; kristina.stavrakeva@mu-plovdiv.bg (K.S.); vesela.kokova@mu-plovdiv.bg (V.K.); 2 Research Institute, Medical University of Plovdiv, 4002 Plovdiv, Bulgaria; iliya.kostadinov@mu-plovdiv.bg; 3 Department of Medical Biochemistry, Faculty of Pharmacy, Medical University of Plovdiv, Vasil Aprilov Str. 15A, 4002 Plovdiv, Bulgaria; ivica.dimov@mu-plovdiv.bg (I.D.); mariya.choneva@mu-plovdiv.bg (M.C.); anelia.bivolarska@mu-plovdiv.bg (A.B.); 4 Department of Pharmacology and Clinical Pharmacology, Faculty of Medicine, Medical University of Plovdiv, Vasil Aprilov Str. 15A, 4002 Plovdiv, Bulgaria; delyan.delev@mu-plovdiv.bg; 5 Department of General and Clinical Pathology, Faculty of Medicine, Medical University of Plovdiv, 4000 Plovdiv, Bulgaria; iliya.bivolarski@mu-plovdiv.bg (I.B.); mariya.koleva@mu-plovdiv.bg (M.K.); 6 Department of Botany and Biological Education, Faculty of Biology, University of Plovdiv “Paisii Hilendarski”, 24 Tsar Assen Str., 4000 Plovdiv, Bulgaria or rumen.mladenov@mu-plovdiv.bg (R.M.); or plamen.stoyanov@mu-plovdiv.bg (P.S.); 7 Department of Bioorganic Chemistry, Faculty of Pharmacy, Medical University of Plovdiv, Vasil Aprilov Str. 15A, 4002 Plovdiv, Bulgaria

**Keywords:** paracetamol-induced hepatotoxicity, animal models, biochemical markers, AST, ALT, MDA, 8-OH-dG, histopathological evaluation, linarin, rosmarinic acid

## Abstract

Acetaminophen (APAP) overdose can result in potentially fatal acute liver failure, with free radical formation identified as a major mechanism of liver tissue damage. *Micromeria frivaldszkyana* (*M. frivaldszkyana*), a rare species endemic to Bulgaria, has demonstrated significant antioxidant activity. Male Wistar rats were treated orally for 7 days with saline; 250, 400, or 500 mg/kg of a water solution of dried methanolic extract of *M. frivaldszkyana*; 100 mg/kg rosmarinic acid (RA); or 125 mg/kg silymarin. Liver toxicity was induced by oral application of 2000 mg/kg APAP on the last day of treatment. Forty-eight hours later, blood and livers were collected for histological and biochemical analysis. The results revealed that treatment with 500 mg/kg of the dried methanolic extract significantly reduced the elevated levels of aspartate aminotransferase, alanine aminotransferase, malondialdehyde, 8-hydroxy-2′-deoxyguanosine, and tumor necrosis factor-alpha in APAP overdose. The present results clearly demonstrate, for the first time, that pre-treatment with methanolic extract of *M. frivaldszkyana* results in significant hepatoprotective effects in the APAP-induced rat model of liver injury. The mechanism of this effect may involve cell membrane protection, decreased lipid peroxidation and DNA damage, and attenuation of aseptic inflammation. These effects can be attributed to the main compounds identified in the extract (linarin, chlorogenic acid, rutin, eupatorin, apigenin, RA).

## 1. Introduction

Paracetamol (acetaminophen, N-acetyl-p-aminophenol, APAP) is a widely used over-the-counter drug available in various formulations. It is commonly used worldwide as an analgesic and antipyretic agent [1,2]. Although considered safe at therapeutic doses, APAP overdose can result in potentially fatal acute liver failure. According to recent reports, nearly 20% of poisoning-related deaths in the United States are attributed to APAP exposure [2].

At therapeutic doses, APAP is primarily metabolized through glucuronidation and sulfation pathways into non-toxic metabolites, which are excreted in the urine [3]. A small fraction undergoes oxidation via cytochrome P450 (CYP450) enzymes (CYP2E1, CYP1A2), resulting in the formation of N-acetyl-p-benzoquinone imine (NAPQI). NAPQI induces oxidative stress, mitochondrial dysfunction, and hepatocellular necrosis through covalent binding to liver proteins [2,4]. NAPQI can be neutralized by conjugation with glutathione (GSH), and the resulting complex is excreted renally. However, in overdose situations, excessive NAPQI production overwhelms GSH capacity, leading to liver damage [1,4].

APAP-induced toxicity is primarily mediated by the generation of free radicals and reactive oxygen species (ROS) [5]. ROS are normally neutralized by antioxidant enzymes such as superoxide dismutase (SOD), catalase (CAT), and glutathione peroxidase (GPx). Depletion of these enzymes is closely associated with tissue damage due to oxidative stress. Additionally, ROS trigger lipid peroxidation, leading to disruption of cell membranes [6]. The latter is indicated by a well-documented increase in the levels of malondialdehyde (MDA) [7]. APAP-driven oxidative stress is also associated with deoxyribonucleic acid (DNA) modifications, notably a marked increase in the levels of 8-hydroxy-2′-deoxyguanosine (8-OH-dG) [8]. Several comprehensive reviews have highlighted the role of inflammation and oxidative cell damage in NAPQI-mediated organ injury, along with the involvement of cytokines such as tumor necrosis factor-alpha (TNF-α) and interleukin-6 (IL-6) [9,10]. Furthermore, APAP hepatotoxicity is directly linked to increased serum levels of the hepatic transaminase enzymes—aspartate aminotransferase (AST) and alanine aminotransferase (ALT) [11]—as well as with hyperbilirubinemia [12].

In recent years, interest in plant-derived compounds with hepatoprotective properties has grown significantly. Furthermore, silymarin, a flavonoid complex extracted from *Silybum marianum* (milk thistle), has a well-documented history of over 50 years in the treatment of liver disorders, including viral hepatitis and alcohol-induced liver injury [13]. Hepatoprotective properties have also been reported for various plant-derived compounds [14], plant extracts [1,15], and even certain mushrooms, such as *Pleurotus ostreatus* [6].

*Micromeria frivaldszkyana* (*M. frivaldszkyana*), a rare plant species of the Lamiaceae family, is endemic to Bulgaria [16]. Scientific data on this species are scarce, and its pharmacological properties remain largely unexplored. Recent studies have described the phytochemical profile of its methanolic extract and assessed its safety in acute and subchronic application in rats [17,18]. The extract demonstrated significant anti-inflammatory activity in a rat paw edema model [17]. Furthermore, a comparative in vitro study involving methanolic extracts from *M. frivaldszkyana*, *M. dalmatica*, *M. juliana*, and *M. cristata* showed that *M. frivaldszkyana* exhibited the highest antioxidant activity in a 2,2′-diphenylpicrylhydrazyl (DPPH) radical scavenging assay [19]. The extract also demonstrated antibacterial activity, notably reducing the growth of *Listeria monocytogenes* ATCC 19111 at a minimum inhibitory concentration (MIC) of 10 mg/mL [16]. Other members of the Lamiaceae family, such as *Stachys germanica* (essential oil), have also been reported to exhibit antioxidant and anti-inflammatory activities, possibly due to the presence of constituents such as bornyl acetate and camphor [20].

In a previous study, ultra-performance liquid chromatography tandem mass spectrometry (UPLC-MS/MS) analysis of *M. frivaldszkyana* methanolic extract led to the identification of 192 compounds. Phenolic acids and flavonoids—primarily as flavonoid glycosides—were the predominant secondary metabolites. Among them, linarin and its derivatives, chlorogenic acid, rutin, eupatorin, and rosmarinic acid (RA) were present in the highest concentrations [17]. These compounds have demonstrated anti-inflammatory and antioxidant properties [21,22,23]. Furthermore, linarin, RA and chlorogenic acid were found effective against liver toxicity in vivo and in vitro [24,25,26].

Based on this evidence, we hypothesize that the methanolic extract of *M. frivaldszkyana* may attenuate liver damage induced by APAP overdose in rats.

The aim of this study is to evaluate the protective effect of orally administered water solution of dried methanolic extract of *M. frivaldszkyana* against APAP-induced liver injury in rats. Organ damage severity was assessed via histological analysis and quantification of serum and tissue levels of specific biomarkers.

## 2. Results

### Effect of Methanolic Extract of M. frivaldszkyana in APAP-Induced Liver Toxicity

**Figure 1:**
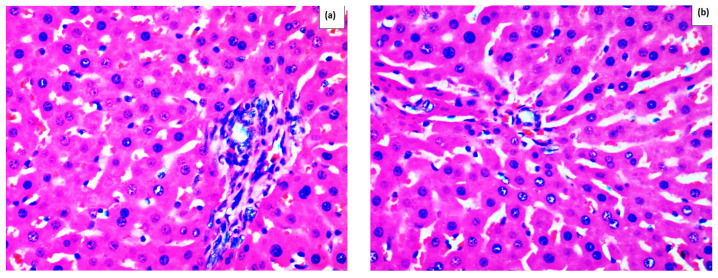

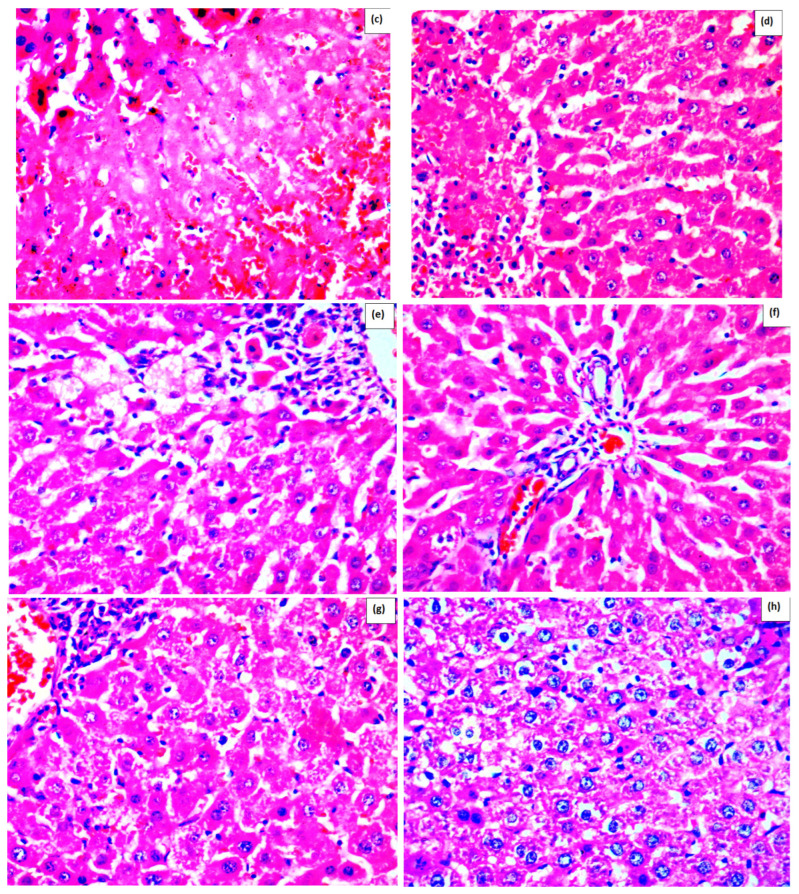
Histological examination of liver tissue. (**a**) group S—control rats, which received saline at 0.1 mL/100 g bw without APAP application; (**b**) group ME500—treated with 500 mg/kg of a water solution of the dried methanolic extract without APAP application; (**c**) group 3 (S+APAP)—saline at a dose of 0.1 mL/100 g and APAP application; (**d**) group 4 (ME250+APAP) rats treated with 250 mg/kg water solution of the dried methanolic extract and APAP application; (**e**) group 5 (ME400+APAP) rats treated with 400 mg/kg water solution of the dried methanolic extract and APAP application; (**f**) group 6 (ME500+APAP) rats treated with 500 mg/kg water solution of the dried methanolic extract and APAP application; (**g**) group 7 (RA+APAP) received 100 mg/kg RA and APAP application; (**h**) group 8 (Sil+APAP) was treated with 125 mg/kg silymarin and APAP application.

As shown in Figure 1, normal liver architecture was observed in the control rats (Figure 1a) and in the rats treated with 500 mg/kg methanolic extract without APAP administration (Figure 1b). The most significant pathological changes were detected in the rats that received an APAP overdose and saline (Figure 1c). In this group (APAP+S), hepatic cell necrosis, dilation of sinusoids, parenchymal hemorrhages, inflammatory and Kupffer cell infiltration, as well as disruption of normal liver architecture were observed. Treatment with the methanolic extract of *M. frivaldszkyana* (Figure 1d–f) reduced the severity of liver damage, however not to the extent observed in rats treated with silymarin (Figure 1h). A reduction in liver damage was also observed in rats treated with RA (Figure 1g); however, the decrease was more pronounced in the groups treated with silymarin and with 500 mg/kg methanolic extract (Figure 1f,h).

In the Control S group, the quantitative analysis showed no evidence of necrosis, sinusoidal dilation, or inflammatory infiltration. Similarly, in the ME500 group, no pathological alterations were detected; necrosis, sinusoidal dilation, and inflammatory infiltration were completely absent (0%). In contrast, the APAP+S group displayed the most severe pathological changes. Necrosis affected approximately 50% of hepatocytes, sinusoidal dilation was markedly increased (80%), and inflammatory infiltration was severe, accompanied by clear disruption of the normal hepatic architecture. In the 250 mg/kg methanolic extract + APAP group, necrosis was observed in 15% of hepatocytes, sinusoidal dilation was present in 30% of the liver tissue, and inflammatory infiltration was moderate. The 400 mg/kg methanolic extract + APAP group showed comparable findings, with necrosis limited to 15%, sinusoidal dilation reduced to 20%, and moderate inflammatory infiltration. The 500 mg/kg methanolic extract + APAP group exhibited results similar to those of the silymarin group. Necrosis was restricted to 8% of hepatocytes, sinusoidal dilation remained at 10%, and inflammatory infiltration was mild. In the RA+APAP group, necrosis was reduced to 18%, sinusoidal dilation reached 20%, and inflammatory infiltration was moderate. In the Silymarin+APAP group, only minimal pathological changes were observed. Necrosis was restricted to 7% of hepatocytes, sinusoidal dilation was limited to 10%, and inflammatory infiltration was mild.

**Figure 2:**
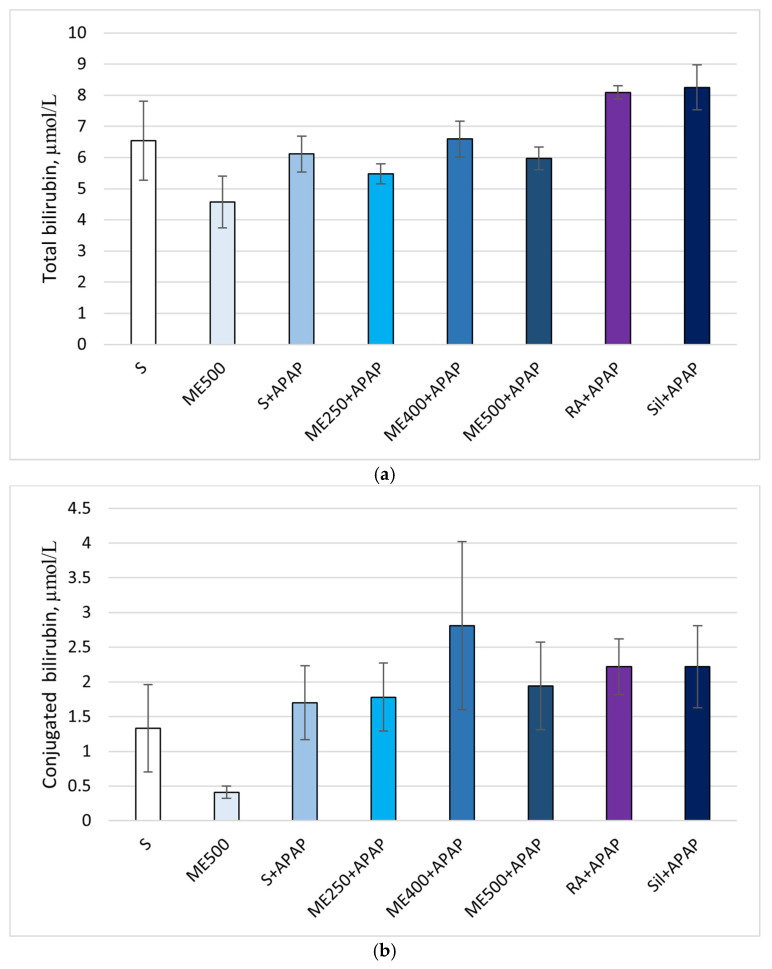

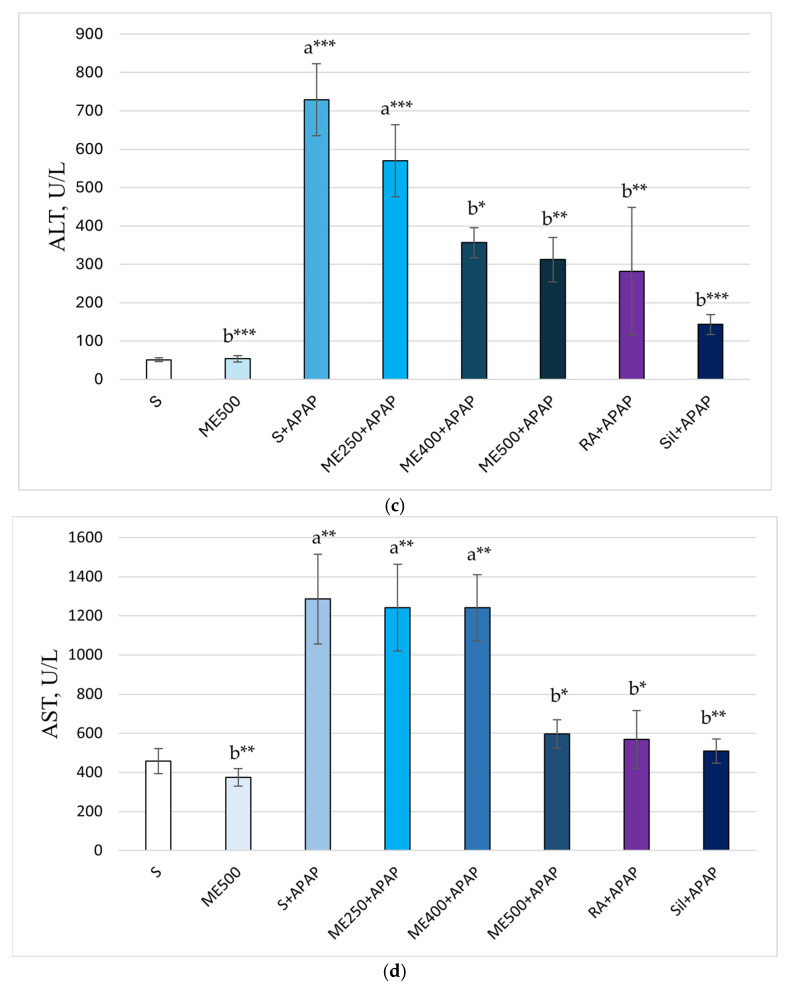
Biochemical markers in serum. (**a**) total bilirubin; (**b**) conjugated bilirubin; (**c**) ALT; (**d**) AST. Data are presented as mean ± SEM. The symbol a ** indicates *p* ≤ 0.01 vs. control group; a *** indicates *p* ≤ 0.001 vs. control group; b * indicates *p* < 0.05 vs. APAP + saline-treated rats; b ** indicates *p* < 0.01 vs. APAP + saline-treated rats; b *** indicates *p* < 0.01 vs. APAP + saline-treated rats, according to one-way ANOVA followed by Tukey’s post hoc test.

No statistically significant changes were observed in total and conjugated bilirubin levels (Figure 2a,b).

Figure 2c shows significantly higher ALT levels in the S+APAP and ME250+APAP groups compared to the control group (728.90 ± 93.94 vs. 50.63 ± 5.07, *p* < 0.001; 569.34 ± 93.82 vs. 50.63 ± 5.07; *p* ≤ 0.001). A significant decrease in ALT was observed in the ME500, ME400+APAP, ME500+APAP, RA+APAP, and Sil+APAP groups compared to the S+APAP group (53.61 ± 8.32 vs. 728.90 ± 93.94, *p* < 0.001; 356.34 ± 39.09 vs. 728.90 ± 93.94, *p* < 0.05; 311.88 ± 58.31 vs. 728.90 ± 93.94, *p* ≤ 0.01; 281.81 ± 165.88 vs. 728.90 ± 93.94, *p* < 0.01; 142.76 ± 25.59 vs. 728.90 ± 93.94, *p* < 0.001).

According to the analysis, a significant increase in serum AST levels was observed in the S+APAP, ME250+APAP, and ME400+APAP groups compared to the saline-treated control group (1284.94 ± 229.22 vs. 457.46 ± 63.08; 1241.21 ± 222.14 vs. 457.46 ± 63.08; 1240.38 ± 169.82 vs. 457.46 ± 63.08, *p* < 0.01). As shown in Figure 2d, serum AST levels were significantly decreased in the ME500, ME500+APAP, RA+APAP, and Sil+APAP groups compared to the S+APAP group (373.84 ± 44.16 vs. 1284.94 ± 229.22, *p* < 0.01; 596.25 ± 72.79 vs. 1284.94 ± 229.22 *p* < 0.05; 568.43 ± 148.36 vs. 1284.94 ± 229.22, *p* < 0.05; 509.03 ± 62.35 vs. 1284.94 ± 229.22, *p* < 0.01).

**Figure 3:**
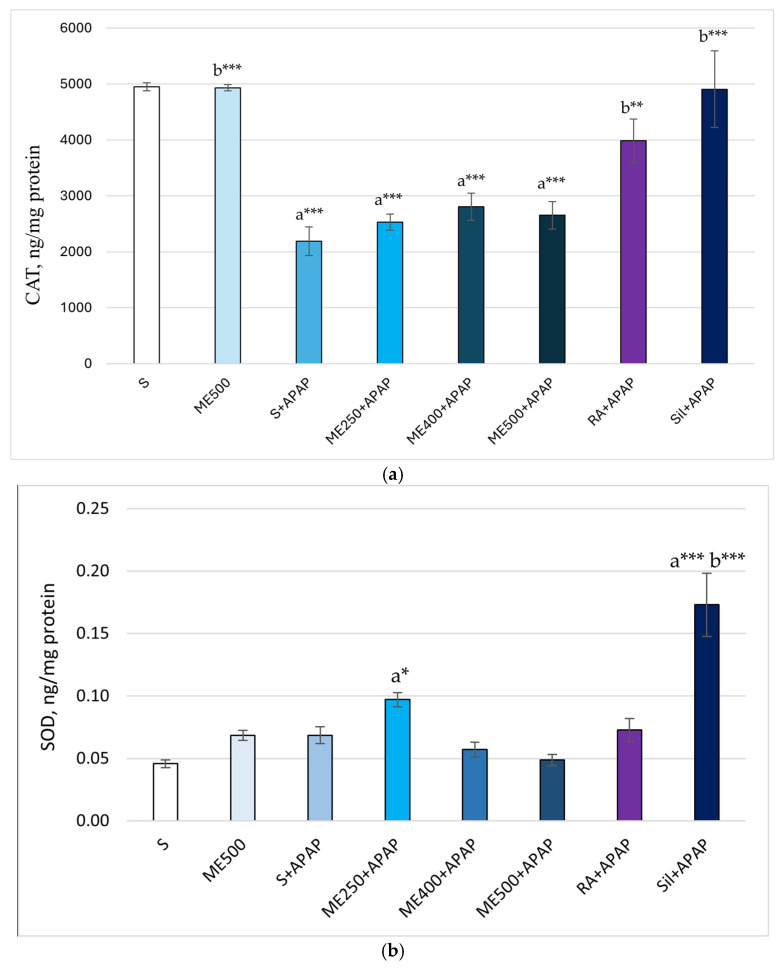

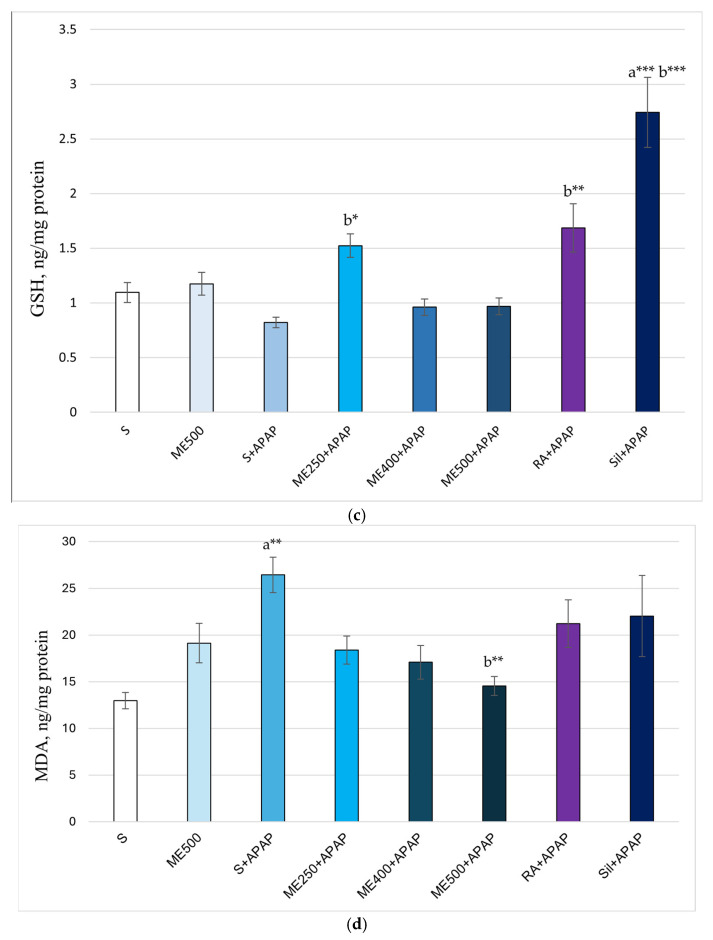

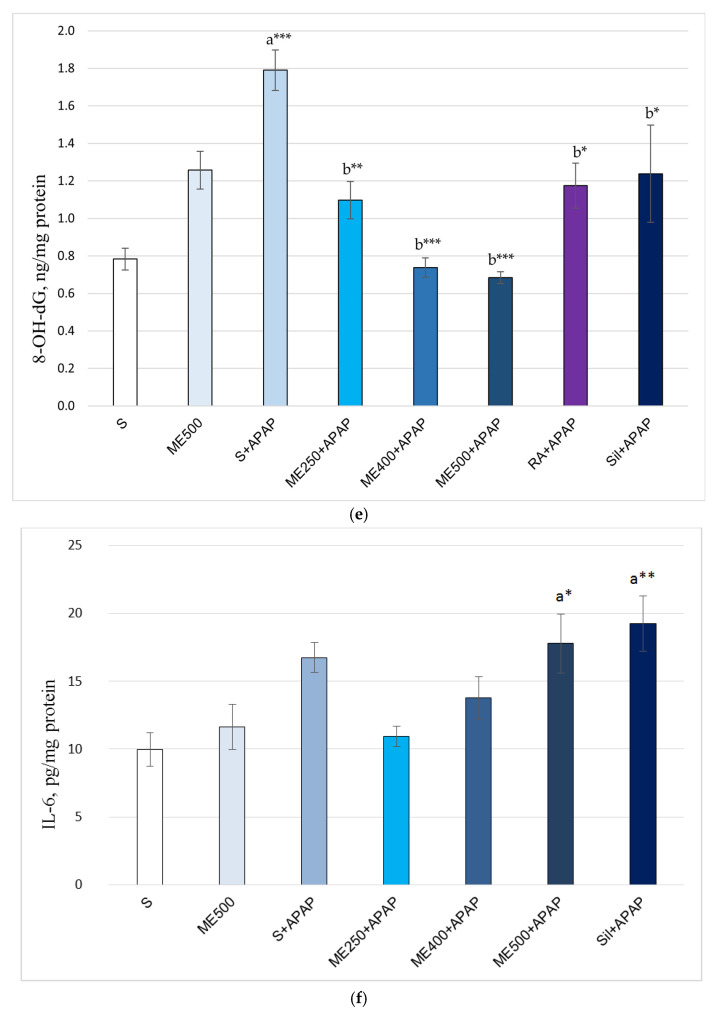

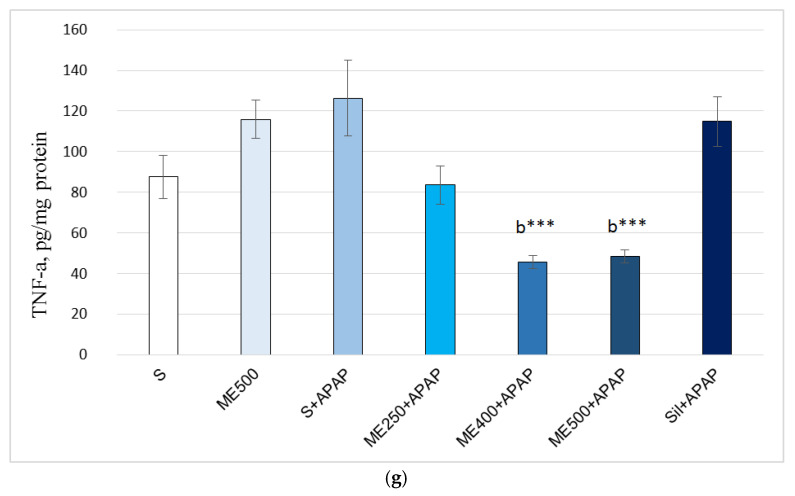
Biochemical markers in liver tissue. (**a**) CAT; (**b**) SOD; (**c**) GSH; (**d**) MDA; (**e**) 8-OH-dG; (**f**) IL-6; (**g**) TNF-α. Data are presented as mean ± SEM. The symbol a * indicates *p* < 0.05 vs. control group; a ** indicates *p* ≤ 0.01 vs. control group; a *** indicates *p* ≤ 0.001 vs. control group; b * indicates *p* < 0.05 vs. APAP+saline-treated rats; b ** indicates *p* < 0.01 vs. APAP+saline-treated rats; b *** indicates *p* < 0.001 vs. APAP+saline-treated rats (One-way ANOVA followed by Tukey’s post hoc test).

Figure 3a demonstrates significantly lower CAT levels in the S+APAP, ME250+APAP, ME400+APAP, ME500+APAP groups compared to the control group (2212.23 ± 254.01 vs. 4951.56 ± 71.55; 2529.66 ± 142.52 vs. 4951.56 ± 71.55, *p* < 0.001; 2807.60 ± 243.82 vs. 4951.56 ± 71.55, *p* ≤ 0.001; 2653.84 ± 245.31 vs. 4951.56 ± 71.55, *p* < 0.001). A significant increase in CAT enzyme levels was observed in the groups treated with ME500, RA and silymarin compared to the S+APAP group (4931. 99 ± 54.77 vs. 2212.23 ± 254.01, *p* < 0.001; 3986.52 ± 387.38 vs. 2212.23 ± 254.01, *p* < 0.01; 4907.14 ± 683.10 vs. 2212.23 ± 254.01, *p* < 0.001).

Elevated SOD levels were observed in ME250+APAP and Sil+APAP groups compared to the control group (0.10 ± 0.006 vs. 0.05 ± 0.003, *p* < 0.05; 0.17 ± 0.025 vs. 0.05 ± 0.003, *p* < 0.001). Additionally, the SOD levels were significantly higher in the Sil+APAP group in comparison to S+APAP (0.17 ± 0.025 vs. 0.07 ± 0.007, *p* < 0.001), as shown in Figure 3b.

Figure 3c shows increased levels of reduced GSH in the Sil+APAP group compared to the controls (2.74 ± 0.32 vs. 1.10 ± 0.09, *p* < 0.001). Significantly higher GSH levels were also observed in groups ME250+APAP, RA+APAP, and Sil+APAP compared to S+APAP (1.52 ± 0.11 vs. 0.82 ± 0.05, *p* < 0.05; 1.68 ± 0.22 vs. 0.82 ± 0.05, *p* < 0.01; 2.74 ± 0.32 vs. 0.82 ± 0.05, *p* < 0.001).

As shown in Figure 3d, serum MDA levels were significantly increased in the S+APAP group compared to the controls (26.42 ± 1.89 vs. 12.97 ± 0.89, *p* < 0.01) and significantly decreased in the ME500+APAP group compared to group S+APAP (14.54 ± 1.02 vs. 26.42 ± 1.89, *p* ≤ 0.01).

Figure 3e illustrates a significant increase in 8-OH-dG levels in the S+APAP group compared to the control rats (1.79 ± 0.11 vs. 0.78 ± 0.06, *p* < 0.001). Significantly lower levels of this marker were found in groups ME250+APAP, ME400+APAP, ME500+APAP, RA+APAP, and Sil+APAP compared to group S+APAP (1.10 ± 0.10 vs. 1.79 ± 0.11, *p* < 0.01; 0.74 ± 0.05 vs. 1.79 ± 0.11, *p* < 0.001; 0.68 ± 0.03 vs. 1.79 ± 0.11, *p* < 0.001; 1.17 ± 0.12 vs. 1.79 ± 0.11, *p* ≤ 0.01; 1.24 ± 0.24 vs. 1.79 ± 0.11, *p* < 0.05.

The changes in the IL-6 levels are shown in Figure 3f. Significantly higher values were observed in the ME500+APAP and Sil+APAP groups in comparison to the controls (17.79 ± 2.16 vs. 9.99 ± 1.23, *p* < 0.05; 19.25 ± 2.05 vs. 9.99 ± 1.23, *p* < 0.01).

Figure 3g demonstrates a significant decrease in TNF-α levels in groups ME400+APAP and ME500+APAP compared to the S+APAP group (45.86 ± 3.21 vs. 126.33 ± 18.54, *p* < 0.001; 48.42 ± 3.16 vs. 126.33 ± 18.54, *p* < 0.001).

## 3. Discussion

The liver injury, which occurs in APAP overdose, is induced by elevated levels of NAPQI. This metabolite is produced through APAP oxidation by CYP450 enzymes and is usually neutralized by GSH. However, excessive production of NAPQI leads to depletion of GSH and liver injury through covalent binding of NAPQI to cellular proteins. Centrilobular hepatocytes are mainly affected by the mechanisms of oxidative stress, mitochondrial dysfunctions, and inflammation, ultimately resulting in cell necrosis [27]. The initial formation of ROS in the cytosol is followed by oxidative stress and the formation of protein adducts in the mitochondria. This induces mitochondrial membrane damage and subsequent hepatic cell necrosis [28]. However, the mechanism of APAP-induced liver injury is more complex, involving the processes of autophagy, sterile inflammation, liver repair, and regeneration [29].

The standard treatment of APAP overdose is based on N-acetylcysteine (NAC) application. NAC is a GSH precursor, which has been used as an antidote for 50 years. By increasing the levels of GSH, NAC reduces NAPQI toxicity and alleviates oxidative stress [30,31]. GSH is the main protective molecule against NAPQI-induced liver damage. In this study, elevated levels of GSH were observed after treatment with RA and silymarin, and similar results were reported by Dkhil et al. (2019) and Yang et al. (2013) [1,32].

The present study revealed a significant increase in the levels of AST and ALT in rats with APAP overdose, which indicates the primary injury of the hepatocyte membranes. Pre-treatment with RA, silymarin, and methanolic extract of *M. frivaldszkyana* reduced the severity of the injury. The optimal results were achieved by silymarin, RA, and the highest dose of the extract. The results are consistent with other authors, who reported decreased levels of hepatic enzymes after treatment with silymarin [1] and RA [7]. Moreover, the results are supported by the histopathological observation, as described in Figure 1. In contrast to Dkhil et al. (2019), we did not register any significant changes in the serum levels of total and conjugated bilirubin [1].

The essential role of oxidative stress in APAP-induced liver toxicity was discussed above and supported by the registered low levels of CAT in the APAP+S group. Silymarin and RA significantly increased this antioxidant enzyme, and these results align with Dkhil et al. (2019) and Yang et al. (2013) [1,32]. Interestingly, the extract of *M. frivaldszkyana* did not induce elevation of SOD or CAT levels in liver tissue. This observation suggests that the hepatoprotective effects of the extract are not mediated via upregulation of cell-protective antioxidant enzymes. However, the involvement of other antioxidant mechanisms cannot be excluded.

The other biomarker related to oxidative stress, MDA, was significantly reduced in rats, who received the highest dose of the extract. Although decreases were also noted at lower doses, they did not reach statistical significance. This suggests reduced lipid peroxidation after treatment with *M. frivaldszkyana* extract. Moreover, suppression of lipid peroxidation is of primary importance for maintaining the normal function of the hepatocyte membrane [15]. This mechanism may explain the observed effect of the extract on the AST and ALT levels. Overall, our results revealed the potential protective effect of the methanolic extract of *M. frivaldszkyana* on the hepatocyte membrane and its hepatoprotective properties in APAP overdose.

As mentioned before, plants from the Lamiaceae family have significant antioxidant activity [16]. Nikolova et al. (2017) performed a DPPH assay on methanolic extracts from various *Micromeria* species (*M. juliana*, *M. cristata, M. frivaldszkyana*, and *M. dalmatica*). The authors observed the highest antioxidant activity in *M. frivaldszkyana* and *M. dalmatica* [19]. More recent studies have further confirmed the strong antioxidant properties of *M. frivaldszkyana* extract. Mladenova et al. (2021) assessed this activity of *M. frivaldszkyana* extract using multiple complementary assays, including DPPH, 2,2-azinobis (3)-ethylbenzthiazoline-6-sulfonic acid (ABTS), ferric reducing antioxidant power (FRAP), cupric ion reducing antioxidant capacity (CUPRAC), and oxygen radical absorbance capacity (ORAC) [16]. Their findings indicated that the ORAC value of *M. frivaldszkyana* (3250.5 ± 208.1 μmol TE/g) is significantly higher than that of many other traditionally used Bulgarian medicinal plants [33].

Oxidation can also occur with DNA, resulting in the formation of 8-OH-dG. Elevated levels of this molecule are used as a marker of oxidative DNA damage. Wang et al. (2015) reported increased 8-OH-dG in rodents after prolonged treatment with APAP [34]. The results of the current study showed a similar increase after a single APAP overdose. A significant decrease was detected in all experimental groups, with the most pronounced effect observed in groups receiving 400 and 500 mg/kg of the extract. In general, the extract induced a significant dose-dependent decrease in this parameter. Based on this, we propose DNA protection as one of the mechanisms underlying the observed liver protection. Moreover, the DNA damage plays an important role in carcinogenesis, which implies further research into the anti-tumor properties of this extract [35].

Excessive ROS production initiates cellular signaling pathways, that upregulate the expression of pro-inflammatory cytokine genes [1]. The APAP-induced hepatocyte necrosis is followed by aseptic inflammation and elevated levels of pro-inflammatory cytokines (e.g., IL-6, IL-1β, TNF-α). Regarding the IL-6 levels, we found no statistical difference between the groups, and our results align with [36]. The decreased levels of TNF-α in the groups treated with 400 and 500 mg/kg methanolic extract of *M. frivaldszkyana* suggested the involvement of the anti-inflammatory properties of the extract in its hepatoprotective effect. Previously, the anti-inflammatory activity of the extract was reported by Stavrakeva et al. (2024) in a model of rat paw inflammation [17]. In contrast to other authors, who reported decreased levels of this cytokine in the same model of APAP-induced liver toxicity after treatment with silymarin, our results did not indicate such differences. This discrepancy may be due to differences in silymarin dosage [1]. The observed reduction in TNF-α levels may be attributed to the high linarin content in the methanolic extract. Ellapan et al. (2022) evaluated the effect of *Ceiba pentandra* extract on carbon tetrachloride (CCl4)-induced liver toxicity. The authors reported decreased TNF-α level in the rats, treated with the extract and explained this effect with the presence of linarin in the ethanolic extract [37]. Similarly, metabolomic analysis of *M. frivaldszkyana* extract identified linarin as the predominant compound [17]. In silico studies have demonstrated a high affinity of linarin for TNF-α converting enzyme. This cytokine is released by Kupffer cells following acute liver injury, which can be induced by hepatotoxic substances challenge [37].

Linarin (also known as acacetin 7-O-rutinoside) is a flavonol glycoside isolated from plant species from the Asteraceae and Lamiaceae families, and genera *Mentha*, *Micromeria*, and *Satureja* are described as particularly rich in linarin [38]. Among the numerous biological activities attributed to this compound, antioxidant, anti-inflammatory, and hepatoprotective properties are of particular interest [24,38]. Linarin has been found effective in CCl4-induced liver toxicity, leading to decreased MDA levels in vitro [24]. The oral administration of some bioactive compounds in vivo may result in low plasma levels due to degradation in the gastrointestinal tract or obstructed absorption by the mucus [39]. However, a pharmacokinetic study showed rapid absorption after oral administration in rats, with appropriate distribution of linarin and its metabolites in liver tissue [40]. This report allows us to hypothesize that the observed hepatoprotective effect may be partially related to the high content of linarin in the extract. However, one should keep in mind that all compounds may contribute to the observed effect, not only those present in the highest amounts [41].

Hepatoprotective properties have also been reported for chlorogenic acid, eupatorin, rutin, apigenin, naringenin, and RA, which are also abundant in the methanolic extract of *M. frivaldszkyana*. Chlorogenic acid (5-caffeoylquinic acid) is a phenolic compound found in significant quantities from coffee, apples, pears, berries, etc. The application of this compound alleviates hepatocellular injury and liver steatosis, reducing serum AST and ALT levels in mice with diet-induced nonalcoholic steatohepatitis [42]. Decreased serum levels of hepatic enzymes by chlorogenic acid were also reported by Hu et al. (2020) in a model of APAP-induced liver injury in mice [43]. In the same model, chlorogenic acid was shown to promote liver regeneration and repair [44]. Additionally, it has been reported to alleviate the hepatic injury in several models of hepatotoxicity by decreasing apoptosis, inflammation, and oxidative injury caused by methotrexate and heavy metals [45]. Increased levels of GSH, SOD, CAT, along with decreased MDA level were reported by Feng et al. (2016) in a model of d-galactose-induced liver toxicity in mice. The authors found a significant decrease in the pro-inflammatory cytokines TNF-α and IL-6 following chlorogenic acid treatment [46].

Eupatorin is another compound with a possible role in the observed effect of the extract. This flavone is a weak acid and undergoes biotransformation to 51 metabolites in rats. Eupatorin demonstrated anti-inflammatory activity in rodent models of paw edema [47].

Rutin (quercetin-3-O-rutinoside) showed hepatoprotective activity in a model of CCl_4_-induced liver toxicity in rats. Decreased mortality and lower serum levels of ALT and AST were observed in pre-treated animals [48]. The hepatoprotective properties of rutin may also be attributed to decreased TNF-α level in rats with deltamethrin-induced hepatotoxicity [49].

Goudarzi et al. (2021) reported restoration of AST, ALT, MDA, and TNF-α levels after apigenin administration in a rat model of methotrexate-induced hepatotoxicity. Treated animals also showed increased levels of SOD, CAT, and GSH compared to those treated only with methotrexate. The authors propose the antioxidant and anti-inflammatory activity of apigenin as a possible mechanism for the observed effects [50].

Similarly, Ahmed et al. (2019) registered reduced serum AST, ALT, and TNF-α levels in rats with APAP-induced liver toxicity after following treatment with naringin and naringenin [51].

Rosmarinic acid, an ester of caffeic acid and 3,4-dihydroxyphenyl lactic acid, is a common compound in many plants, and its biological effects are widely explored. Antibacterial, antioxidant, and anti-inflammatory properties are documented in vivo and in vitro [52]. This phenol carboxylic acid has shown hepatoprotective activity in a wide spectrum of experimental models, including liver injury induced by diabetes, CCl_4_, and tert-butyl hydroperoxide (t-BHP) [53]. In a rat model of APAP-induced hepatotoxicity, RA in a dose of 100 mg/kg significantly reduced the elevated levels of AST, ALT, MDA, while increasing hepatic GSH levels [7]. In this study, we detected the same changes in AST, ALT, and GSH. Although MDA levels decreased in the RA-treated group, the reduction did not reach statistical significance, which may be attributed to differences in the experimental protocol. As mentioned above, RA was reported as one of the compounds present at the highest concentrations in the methanolic extract of *M. frivaldszkyana* [17]. We included a group treated with RA to evaluate the similarity between the effects of RA and the extract. Table 1 compares the highest dose of the extract with the two positive controls (RA and silymarin). The changes in the main biomarkers of liver function (AST and ALT) induced by the extract and RA are identical, and the same applies to the levels of SOD. These results allow us to speculate that the abundance of RA in the methanolic extract may be responsible for some of the observed effects. However, in the extract, all components act synergistically and this hypothesis is further supported by the different results obtained for the two groups with respect to CAT, GSH, and MDA levels (Table 1).

Based on the current results, we can speculate that the mechanism of the observed hepatoprotective effect could be attributed to cell membrane protection, decreased lipid peroxidation and DNA damage, and attenuation of aseptic inflammation. However, the mechanism of APAP-induced liver toxicity is more complex and involves the formation of peroxynitrite and adducts of NAPQI to mitochondrial proteins, as well as an influence on signaling pathways with an essential role in cell survival and aseptic inflammation [1]. The present study sheds light on only a limited aspect of the mechanisms potentially modulated by the methanolic extract of *M. frivaldszkyana*. The contribution of additional pathways cannot be excluded and will be the subject of future investigations. Given the strong antioxidant properties of the extract, investigation of its effectiveness against other animal models of liver toxicity, as well as other organs, is also in the scope of our future research.

### Limitations and Future Directions

The following limitations apply to this study: only male Wistar rats were used in the experiment; using a different sex, strain, or route of administration may yield different outcomes. The experiment was performed after 7 days of application of the extract, and longer pre-treatment may have different effects. The APAP toxicity was induced by a single overdose, and the outcome may differ in the case of chronic APAP administration. The present study is limited to exploring the effects of the methanolic extract of *M. frivaldszkyana* in APAP-induced overdose in rats. The specific pathways and target molecules remain to be elucidated and are beyond the scope of the current experiments. IL-6 and TNF-α levels were measured in the groups S, ME500, S+APAP, ME250+APAP, ME400+APAP, ME500+APAP, and Sil+APAP. The RA+APAP group was excluded due to insufficient biological material. In-depth mechanistic studies and extract standardization are necessary to ensure future translational potential.

## 4. Materials and Methods

The experiments were performed according to the following permissions: (1) permit № 352/30 May 2023 by the Bulgarian Food Safety Agency; (2) protocol № 6/05 October 2023 by the Ethics Committee of the Medical University, Plovdiv, Bulgaria; (3) permit № 996/09 August 2023 by the Ministry of Environment and Water for plant material. The study protocol was developed and performed in alignment with the ARRIVE guidelines, the EU Directive 2010/63/EU on animal experiments, and the relevant national and institutional regulations.

### 4.1. Chemicals and Reagents

Methanol (≥99.8%, cat. No. 179337), APAP (≥98.0%, batch No. MKCS3304), RA (≥96.0%, batch No. BCCJ6033), and silymarin (≥30.0%, batch No. BCCH4151) were obtained from Merck SA (Germany). Eosin Y (1% aqueous solution, cat. № 294/EOY-10-OT-2.5L), formaldehyde 4% (10% neutral buffer, cat. № 294/FNB4-10L), Histanol 100 (cat. № 294/H100-5L), Histanol 95 (cat. № 294/H95-5L), hematoxylin G3 (cat. № 294/HEMG3-OT-2.5L), acetone (cat. № 48/3413/5) and xylol (cat. № 348/3410/20) were obtained from BIOCARE Medical (Pacheco, CA, USA).

### 4.2. Plant Material and Preparation of the Methanolic Extract

The aerial parts of *M. frivaldszkyana* (approximately 800 g of fresh biomass) were harvested during the 2023–2024 vegetation season from the Bulgarka Nature Park, located in the Middle Stara Planina floristic region, in accordance with previously established protocols [17,18]. The herbarium of the Agricultural University in Plovdiv (SOA) houses the voucher specimen (accession № 062648). The plant material was dried for 10 days in a shaded area at a controlled ambient temperature of 22 ± 2 °C. Drying was deemed complete when leaf stalks fractured upon bending and the petioles crumbled easily under pressure. Following this, the dried material was milled to a fine powder with an average particle size below 400 μm using a GRINDOMIX GM200 laboratory mill (RETSCH GmbH, Haan, Germany).

To prepare the methanolic extract, 10 g of the powdered sample was macerated in 70% methanol (1:10 *w*/*v*) for 24 h at room temperature (22 ± 2 °C) with continuous stirring in a light-protected container. To improve extraction efficiency, the sample underwent ultrasonication in three 15-min cycles at 30 °C. The next step included centrifugation at 6000× *g* for 15 min, and filtration of the supernatant using Whatman No. 1 filter paper (Sigma-Aldrich, Burlington, MA, USA). The residual plant material was subjected to the same extraction process twice more. The solvent was removed from the combined extracts using a rotary evaporator (Heidolph, Schwabach, Germany) under reduced pressure at 50 °C [17,18]. The extraction yield was 54.8% of the initial dry plant material, corresponding to 5.48 g of dried extract.

### 4.3. Animals and Treatment

A total of 56 male Wistar rats (body weight 210–260 g) were used in the study. They were randomly assigned to eight groups, each comprising seven animals. The animals were maintained under standard laboratory conditions, including a temperature of 22 ± 1 °C, relative humidity of 45%, and a 12-h light/12-h dark cycle, with unrestricted access to food and water.

We followed the protocol for APAP-induced liver toxicity described by Ahmed et al. (2023) [4]. After a one-week acclimatization period, the animals were treated orally for 7 consecutive days according to the following group assignments:

Groups 1 (S, control) and 3 (S+APAP) received saline at a dose of 0.1 mL/100 g body weight (bw); group 2 (ME500) received 500 mg/kg of a water solution of the dried methanolic extract; groups 4, 5, and 6 (ME250+APAP, ME400+APAP, ME500+APAP) were treated with 250, 400, and 500 mg/kg water solution of the dried methanolic extract, respectively; group 7 (RA+APAP) received 100 mg/kg RA; group 8 (Sil+APAP) was treated with 125 mg/kg silymarin.

On the evening of day 6, all animals were fasted for 12 h with free access to water. On day 7, the groups S+APAP, ME250+APAP, ME400+APAP, ME500+APAP, RA+APAP, and Sil+APAP received 2000 mg/kg of APAP via gastric gavage. Three hours later, the respective treatment (extract or compound) was applied to each group. Animals were euthanized 48 h after APAP administration. Blood and liver samples were collected. Portions of each organ were fixed for histopathological analysis, while the remaining tissue was stored at −80 °C and later homogenized for biochemical evaluation.

The duration of pre-treatment prior to APAP administration was selected based on previous studies evaluating the protective effects of plant extracts and natural compounds in APAP-induced liver injury models [1,7,54]. The dose and duration of APAP administration were also based on a review of the literature. The methanolic extract doses were determined according to recommendations by Hanafy et al. (2016), which suggest using 1/10 and 1/20 of the estimated LD_50_ for pharmacological testing [55]. In our previous study, we demonstrated the safety of *M. frivaldszkyana* extract at single oral doses up to 5000 mg/kg [17]. The intermediate dose of 400 mg/kg was included to better characterize the extract’s dose-dependent effects.

The dose of RA was selected based on the study by Hasanein et al. (2017), which demonstrated its beneficial effect in the APAP-induced liver toxicity model [7]. Silymarin, a well-known hepatoprotective agent, was used as a positive control and its dose was also selected based on published data [56].

#### 4.3.1. Histopathological Observation

The experimental protocol was based on the description of other authors [57,58,59] and was reported previously [18].

Immediately following collection, the organs were placed in a 10% neutral-buffered formalin solution to ensure proper fixation. The fixed samples were subsequently processed and embedded in paraffin blocks. The entire histological preparation included the following steps:

1. Tissues were immersed in formalin for 24 h, thoroughly rinsed, and then soaked in distilled water for 30 min to eliminate any residual fixative.
2. Dehydration was achieved through progressive immersion in 95% and then 100% ethanol (Histanol^®^ 100%), each step lasting 4–5 h.
3. After dehydration, the tissues were treated with acetone for 20 min, followed by xylol for 30 min. They were then embedded in molten paraffin and formed into blocks. Thin slices, 5 μm in thickness, were obtained from these blocks and stained with hematoxylin and eosin for microscopic examination.

Histopathological analysis was performed independently by two pathologists who were blinded to the experimental groups. Morphological alterations in the tissues from treated groups were compared to controls. Leica DM500 (Leica Microsystems GmbH, Wetzlar, Germany) and Zeiss AXIO Scope A1 (Carl Zeiss Microscopy GmbH, Jena, Germany) microscopes were used for sample observation.

Liver samples were examined for pathological changes such as architectural disruption, portal and lobular inflammation, sinusoidal dilation and congestion, granuloma formation, necrosis, degeneration, and steatosis. Tissue pathology was classified according to the Roenigk scoring system (Table 2).

#### 4.3.2. Preparation of Liver Homogenates and Evaluation of Tissue-Specific Toxicity Markers

On the day of the analysis, the frozen liver tissues were brought to room temperature and homogenized in a lysis 0.1 M PBS buffer with added Triton 100× (1:9 *w*/*v*), pH 7.4 [61] on a Polytron mechanical homogenizer (KINEMATICA, Malters, Switzerland). The obtained whole-liver homogenates were afterwords centrifuged at 10,000 rpm for 10 min at 4 °C using MPW-352R centrifuge (Warsaw, Poland) [62] and the resultant supernatant was used for the assessment of the following parameters: (1) oxidative cell damage and antioxidant protection markers—MDA, 8-OH-dG, CAT, SOD1, and GSH; (2) pro-inflammatory cytokines—IL-6 and TNF-α, all measured in the supernatant of whole-liver homogenate. All listed parameters were analyzed via an enzyme-linked immunosorbent assay (ELISA) method on an ELISA microplate reader (HumanReader, HUMAN, Wiesbaden, Germany) using commercial kits [ELISA Kit for CAT, Cloud-Clone Corp., Katy, TX, USA, Cat.No. SEC418Ra; MDA ELISA Kit, Cat.No. E-EL-0060; 8-OH-dG ELISA Kit, Cat.No. E-EL-0028; SOD1 (Soluble) ELISA Kit, Cat.No. E-EL-R1424; GSH ELISA Kit, Cat.No. E-EL-0026; Rat IL-6 ELISA Kit, Cat.No. E-EL-R0015; Rat TNF-α ELISA Kit, Cat.No. E-EL-R2856, Elabscience Biotechnology Inc., Houston, TX, USA]. All assays were carried out using ELISA kits from the same production lot in order to minimize batch-to-batch variability, and internal controls supplied by the manufacturer were included on each plate to ensure reproducibility.

#### 4.3.3. Biochemical Markers in Serum

Whole blood was collected from the animals into VACUTEST KIMA tubes (Arzergrande, Italy) and left at room temperature until visible clot formation. The samples were then centrifuged in an MPW-352R unit (MPW Med. Instruments, Warsaw, Poland) at 3000 rpm for 10 min at 4 °C. The resulting serum was separated, aliquoted, and stored at −80 °C (ULT C200) until analysis. It was used for spectrophotometric assessment of the levels of the following markers for liver toxicity: AST (HUMAN Diagnostics GmbH, Wiesbaden, Germany, Ref. No. 12021), ALT (HUMAN Diagnostics GmbH, Wiesbaden, Germany, Ref. No. 12022), total bilirubin (HUMAN Diagnostics GmbH, Wiesbaden, Germany, Ref. No. 10746), and conjugated bilirubin. Biochemical measurements were performed on an Evolution 300 UV-Vis spectrophotometer (Thermo Fisher Scientific, Waltham, MA, USA) following the protocols supplied with the commercial kits.

### 4.4. Statistical Analysis

Statistical processing of the data was performed with SPSS software, version 17.0 (IBM, Armonk, NY, USA). Results are presented as mean ± SEM. Differences between group means were evaluated by one-way analysis of variance (ANOVA) and Tukey’s post hoc test. Statistical significance was defined as *p* ≤ 0.05.

## 5. Conclusions

The present results clearly demonstrate, for the first time, that pre-treatment with methanolic extract of *M. frivaldszkyana* for 7 days results in significant hepatoprotective effects in an APAP-induced rat model of liver injury. The mechanism of this effect could be attributed to cell membrane protection, decreased lipid peroxidation and DNA damage, and attenuation of aseptic inflammation. These effects can be attributed to the main compounds identified in the extract (linarin, chlorogenic acid, rutin, eupatorin, apigenin, RA).

## Figures and Tables

**Table 1:** A comparison of the effects of ME500, RA, and silymarin application on biochemical markers in rats with a model of APAP-induced liver toxicity ^1^.

Marker	ME500+APAP	RA+APAP	Sil+APAP
Total bilirubin	0	0	0
Conjugated bilirubin	0	0	0
ALT	-	-	-
AST	-	-	-
CAT	0	+	+
SOD	0	0	+
GSH	0	+	+
MDA	-	0	0
8-OH-dG	0	0	0
IL-6	0	n/a	0
TNF-α	-	n/a	0

^1^ “+” indicates increased levels, “-“ indicates decreased levels, and “0” indicates no statistical changes in comparison to the group treated only with APAP (group S+APAP); n/a—no available data.

**Table 2:** Roenigk classification (adopted from Berends et al. (2007) [60]).

Grade ^1^	Accumulation of Fats	Nuclear Pleomorphism	Fibrosis	Necrosis/Inflammation
1	Mild or none	Mild or none	None	With or without mild portal inflammation
2	Moderate or severe	Moderate or severe	None	Moderate or severe portal inflammation
3a	With or without	With or without	Mild (fibrosis extending into acini)	With or without
3b	With or without	With or without	Moderate or severe	With or without
4	With or without	With or without	Cirrhosis	With or without

^1^ Grade 1—mild toxicity; Grade 2—moderate toxicity; Grade 3a—severe toxicity; Grade 3b or 4—indication for discontinuation of the treatment.

## Data Availability

The data presented in this study are available on request from the corresponding author.

## Abbreviations

The following abbreviations are used in this manuscript:

8-OH-dG	8-hydroxy-2′-deoxyguanosine
ABTS	2,2-azinobis (3)-ethylbenzthiazoline-6-sulfonic acid
ALT	Alanine aminotransferase
APAP	Acetaminophen (paracetamol)
AST	Aspartate aminotransferase
CAT	Catalase
CCL_4_	Carbon tetrachloride
CUPRAC	Cupric ion reducing antioxidant capacity
CYP	Cytochrome P450
DNA	Deoxyribonucleic acid
DPPH	2,2′-diphenylpicrylhydrazyl
ELISA	Enzyme-linked immunosorbent assay
FRAP	Ferric reducing antioxidant power
GPx	Glutathione peroxidase
GSH	Glutathione
IL-6	Interleukin-6
MDA	Malondialdehyde
MIC	Minimum inhibitory concentration
NAC	N-acetylcysteine
NAPQI	*N*-acetyl-*p*-benzoquinone imine
ORAC	Oxygen radical absorbance capacity
RA	Rosmarinic acid
ROS	Reactive oxygen species
SOD	Superoxide dismutase
t-BHP	Tert-butyl hydroperoxide
TNF-α	Tumor necrosis factor-alpha
UPLC-MS/MS	Ultra-performance liquid chromatography tandem mass spectrometry
